# The Preparation, Characterization, Mechanical and Antibacterial Properties of GO-ZnO Nanocomposites with a Poly(l-lactide)-Modified Surface

**DOI:** 10.3390/ma11020323

**Published:** 2018-02-23

**Authors:** Mingwei Yuan, Chengdong Xiong, Lin Jiang, Hongli Li, Minglong Yuan

**Affiliations:** 1 Engineering Research Center of Biopolymer Functional Materials of Yunnan, Yunnan Minzu University, Kunming 650500, China; yuanmingwei@163.com (M.Y.); jianglin_1981@163.com (L.J.); honglili1982@163.com (H.L.); 2 Chengdu Institute of Organic Chemistry, Chinese Academy of Sciences, Chengdu 610041, China; xiongcdcioc@163.com

**Keywords:** GO-ZnO-PLLA, composite materials, nano-ZnO, antibacterial performance, mechanical properties

## Abstract

Graphene oxide (GO) was employed for the preparation of GO-zinc oxide (ZnO). The hydroxyl group on the surface was exploited to trigger the l-lactide ring-opening polymerization. A composite material with poly(l-lactide) (PLLA) chains grafted to the GO-ZnO surface, GO-ZnO-PLLA, was prepared. The results demonstrated that the employed method allowed one-step, rapid grafting of PLLA to the GO-ZnO surface. The chemical structure of the GO surface was altered by improved dispersion of GO-ZnO in organic solvents, thus enhancing the GO-ZnO dispersion in the PLLA matrix and the interface bonding with PLLA. Subsequently, composite films, GO-ZnO-PLLA and GO-ZnO-PLLA/PLLA, were prepared. The changes in interface properties and mechanical properties were studied. Furthermore, the antibacterial performance of nano-ZnO was investigated.

## 1. Introduction

The increasing severity of global white pollution caused by food packing materials, in addition to the petroleum crisis, has promoted studies on green and eco-friendly food packaging materials that are renewable, degradable, and non-toxic. To date, there have been breakthroughs in the field. Green and renewable polymers such as polylactide (PLLA), polyglycolic acids, and polyamino acids have been developed and widely applied in food packaging research. Among them, PLLA is the material with the best applicability and is recognized as a material that can contribute to the green material world of the 21st century [1]. The use of green, renewable, and degradable materials in rePLLAcing conventional petroleum PLLAstics is an effective means to further ensure food safety, environmental protection, and resource conservation; it is also the trend of technical development and the goal of scientists. Inorganic nanomaterial-PLLA composite films can potentially be developed as next-generation food-packaging materials. To apply PLLA in food packaging, the water and gas barrier properties of PLLA must be enhanced, especially for modified atmosphere packaging used in meat packing, which requires a more effective gas barrier than regular food. Recently, inorganic nanoparticles and nanowires have been applied to modify materials such as PLLA and solve their barrier issues. Additional research and review articles [2,3,4,5,6] have reported the use of nanomaterials such as zinc oxide (ZnO), titanium dioxide, hydroxyapatite, silicate, silver complexes, smectite, nanoclays, and carbon nanotubes. The modification approaches include solution blending, melt blending, and coating. The results have indicated that the use of nanomaterials yields effective improvements to or solutions for water vapor and gas barriers, as well as the heat resistance of the PLLA materials. Some nanomaterials such as ZnO-PLLA nanocomposite materials may even improve anti-UV and antibacterial properties and show characteristics for active food packaging [7,8,9,10]. There have been many studies on applications of inorganic nanomaterial-PLLA composite films in packaging of different types of food. Many scientists, based on these studies, are optimistic that inorganic nanomaterial-PLLA composite films are the new generation of green food packaging material. In recent years, graphene and GO have been utilized in PLLA nanocomposites, and it is found that expanded graphite or GO can increase the crystallinity and the heat-resistant temperature of the nanocomposite [11,12,13,14,15]. Feng et al. developed a versatile route by grafting polymer on the GO to enhance the properties of nanocomposites [16,17]. In our study, we grafted PLLA on the graphene oxide (GO)-ZnO compounds to prepare nanomaterial graphene oxide (GO)-ZnO-poly(l-lactide), and to modify PLLA using this nanomaterial, which is structurally similar to PLLA but is surface-modified by low-molecular-weight PLLA. A high-barrier nano-PLLA film material was prepared, providing a new paradigm and a new method to solve a critical issue encountered in the applications of nano-PLLA composite materials in food packaging, as well as a foundation with theoretical support for studies on next-generation green food packaging materials. 

## 2. Materials and Methods

### 2.1. Materials

#### 2.1.1. Preparation of Raw Materials

GO was provided by Suzhou Carbon Abundance Graphene Science and Technology Co., Ltd. (Suzhou, China) with the following specifications: purity of approximately 99 wt %, thickness of 0.6–1.0 nm, diameter of 0.5–5 μm, in 1–2 layers, and a specific surface area of 1000–1217 m^2^/g. l-lactide was provided by PURAC. Grade 4032D, from NatureWorks (Minnetonka, MN, USA) was employed.

#### 2.1.2. Preparation of GO-ZnO-PLLA

**Scheme 1:**
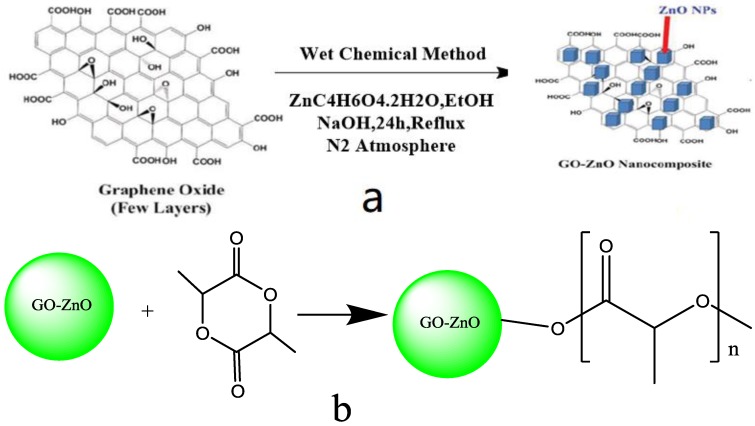
Synthetic route of (**a**) GO-ZnO and (**b**) GO-ZnO-PLLA.

The GO-ZnO was prepared according to the literature [18]. Graphite oxide was dispersed in ethanol (2 mg/1 mL) and sonication for 1 h under ambient conditions. Subsequently, 0.880 g of zinc acetate (ZnC_4_H_6_O_4_·2H_2_O) was dissolved into the mixture while stirring. Then, a predetermined amount of NaOH solution was added to the mixture and pH of the solution was adjusted to 10, after being stirred for 30 min. The mixture was then transferred to a 100mL round bottom flask and heated to 140 °C under N2 atmosphere for 24 h. The prepared composites were then centrifuged and washed by distilled water for several times.The product was dried in a vacuum oven for 24 h at 60 °C. A simple chemical approach was employed for the GO-ZnO nanocomposite in ethanol medium, as shown in Scheme 1a. In 1000-mL round-bottom flasks, 800 mL of 1,4-dioxane was added along with 0.4 g of GO-ZnO (GO-ZnO was prepared according to the literature). The mixture was sonicated for 1.5 h to form an evenly dispersed brown suspension. The suspension was infused with 80 g of l-lactide, stirred, heated under reflux, dissolved until clear, and slightly cooled. The solvent was evaporated under atmospheric pressure at 120 °C. Stannous octoate (0.08 g) was added to the solution, followed by a temperature increase to 140 °C for a 1 h polymerization reaction. Reagents were determined to be polymerized if the mixture could not be stirred. The reaction time was extended for 1 h, and the mixture was then cooled to room temperature. Then, chloroform was used to dissolve the polymerization products. At room temperature for 12 h, the insoluble substances were removed by centrifugation. The filtrate of homogeneous phase was precipitated by excessive ethanol to achieve solid. The solid were filtered and dried under vacuum at 60 °C for 8 h. The product gained was GO-ZnO-PLLA. The reaction equation is shown in Scheme 1b. As a contrast, PLA and GO-ZnO are also dissolved in chloroform, and at room temperature for 12 h, the insoluble substances were removed by centrifugation. The filtrate of homogeneous phase was precipitated by excessive ethanol to achieve solid. The solid were filtered and dried under vacuum at 60 °C for 8 h. The product gained was not GO-ZnO/PLLA blend compound, it was PLA. Filtration after centrifugation was dried by freeze-drying method to obtain GO-ZnO. 

#### 2.1.3. Preparation of the Composite Material GO-ZnO-PLLA/PLLA

Samples of 4, 8 and 12 g of GO-ZnO-PLLA were obtained and mixed separately in 2000 mL of chloroform. The mixtures were mechanically stirred until dissolved to yield a clear solution, followed by 2 h of ultrasonication. The mixture was then precipitated by 5000 mL of anhydrous ethanol to yield gray-whitish solids. The solids were dried under vacuum at 60 °C for 8 h. The products gained were Go-ZnO-PLLA/PLLA composite materials containing 0.5%, 1%, and 1.5% GO-ZnO-PLLA.

#### 2.1.4. Preparation of the GO-ZnO-PLLA/PLLA Composite Film

The composite material obtained above of Go-ZnO-PLLA/PLLA, using the technology of streaming film. Flow film testing machine from Shanghai kechuang plastic products co. LTD (Shanghai, China), Models for LY-300. The thickness of the preparation was 0.02 mm. Film casting equipment was employed at temperatures of 160, 170, 175, 170, 170, and 165 °C for zones 1, 2, 3, 4, 5, and 6, respectively, at a rotation speed of 100 r/min.

## 3. Results and Discussion

### 3.1. Characterization of GO-ZnO-PLLA

In our experiment with the preparation of GO-ZnO-PLLA, the polymerization was completed. Then chloroform was used to dissolve the polymerization products. After 12 h of placement, we found that the chloroform solution was homogeneous. No insoluble substance was found by centrifugation. The filtrate of homogeneous phase was precipitated by excessive ethanol to achieve solidity. The solids were filtered and dried under vacuum to obtain GO-ZnO-PLLA. In the comparison experiment of preparing GO-ZnO/PLLA blend compound, we found that the chloroform dissolved in GO-ZnO/PLLA after 12 h statics was divided into two phases. Because GO-ZnO is insoluble in chloroform, it will be precipitated from the solution. After centrifuge separation, the insoluble substance obtained is pure GO-ZnO. The compounds dissolved in chloroform were found to be pure PLA after the same process. The two experiments also showed that free GO-ZnO could be separated from GO-ZnO-PLLA or PLLA by chloroform dissolution and centrifugation. If GO-ZnO/PLLA blend compound is to be prepared, the GO-ZnO and PLA cannot be centrifuged after mixing in chloroform, GO-ZnO/PLLA can be obtained by direct freeze-drying.

**Figure 1:**
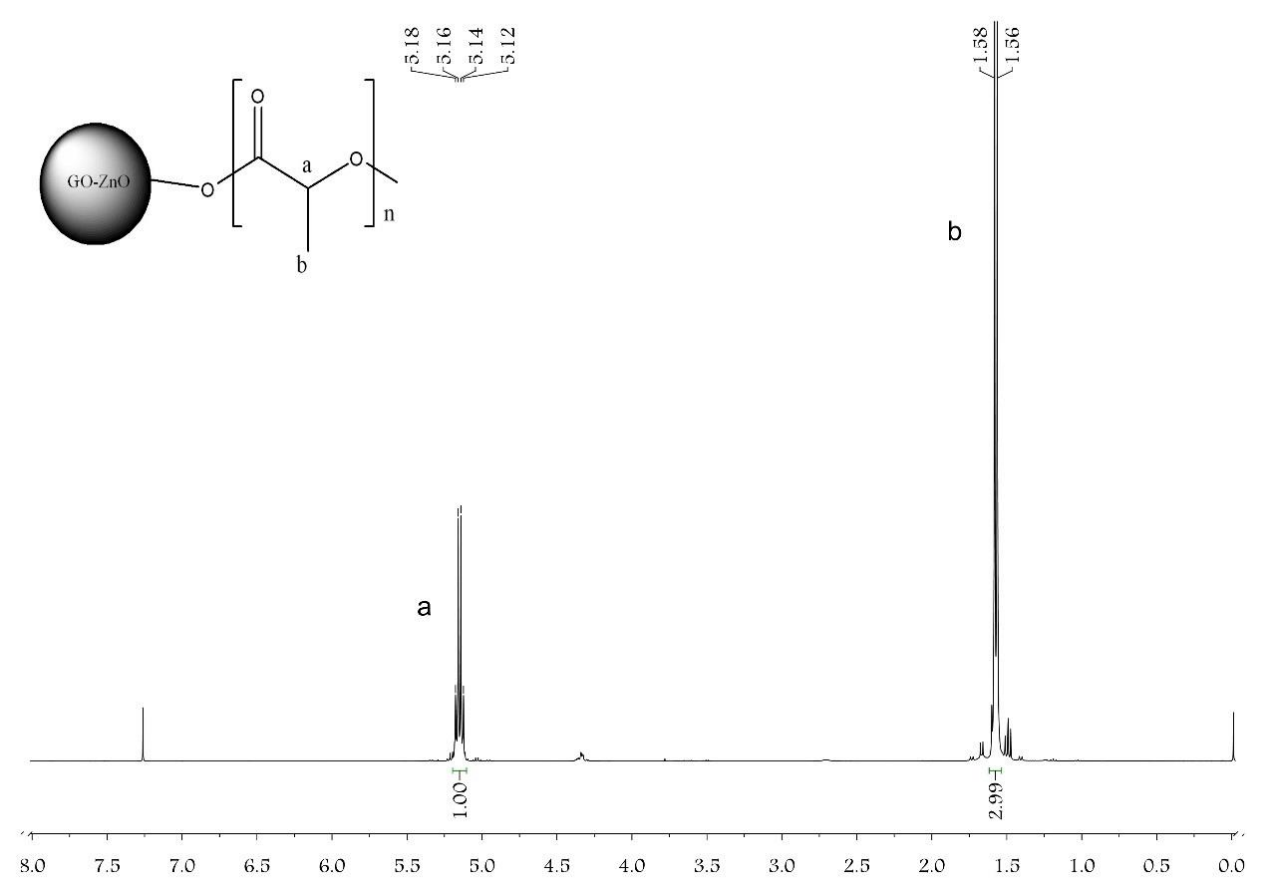
400 MHz ^1^H NMR spectrum of the GO-ZnO-PLLA sample.

To confirm that the GO-ZnO-PLLA has a PLA structure unit, we performed magnetic characterization on the GO-ZnO-PLLA polymers. As seen in Figure 1, the characteristic peaks of PLLA occurred at 1.5 and 5.1 ppm, which is consistent with information reported in the literature [19,20,21,22,23,24]. These characteristic peaks arise from the protons of the methyl (a) and the methenyl (b) groups on the PLLA chain illustrated in the chemical structure in Figure 1. As seen in the nuclear magnetic resonance (NMR) spectrum, the hydroxyl group on the GO-ZnO surface should be successfully triggered by the l-lactide ring-opening polymerization. 

**Figure 2:**
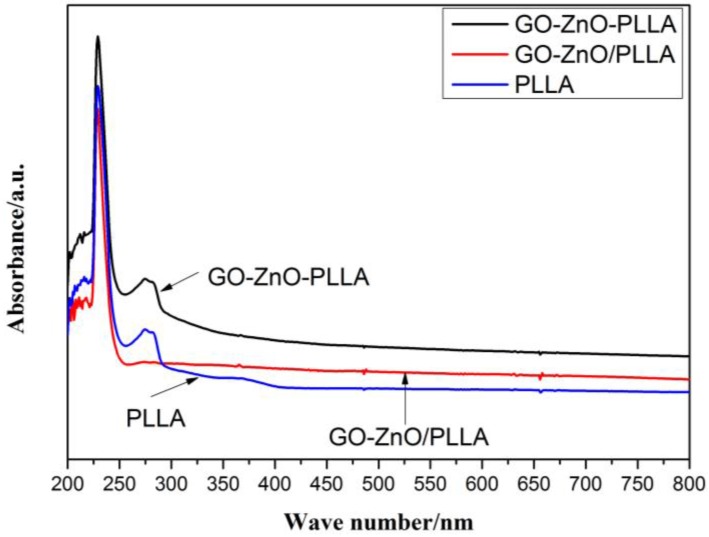
UV/Vis spectra of PLLA, GO-ZnO-PLLA, and GO-ZnO/PLLA.

To confirm that the GO-ZnO-PLLA has a GO-ZnO structure unit, we performed magnetic characterization on the GO-ZnO-PLLA polymers. Figure 2 shows the UV/Vis spectra of PLLA, GO-ZnO-PLLA and GO-ZnO/PLLA. The UV/Vis spectrum of PLLA, GO-ZnO-PLLA and GO-ZnO/PLLA was observed in the DCM solution, while that of PLLA, GO-ZnO-PLLA and GO-ZnO/PLLA was in the solution. GO-ZnO-PLLA shows very broad absorption with continuously decreasing intensity ranged from 220 to 330 nm. On the other hand, PLLA and GO-ZnO-PLLA shows the absorption in the range from 250 to 330 nm, and no absorption peak is observed in the 330 to 800 nm range. Further, PLLA, GO-ZnO-PLLA and GO-ZnO/PLLA shows characteristic peaks in the wave length region shorter than 250 nm, while no evident absorption in the higher wave length region is shown. In the absorption spectrum in the range from 220 to 320 nm, the GO-ZnO-PLLA shows absorption with special features characteristics for both PLLA, indirectly indicating that the PLLA chain was grafted onto the surface of GO-ZnO [25].

### 3.2. GO-ZnO-PLLA Molecular Weight Measurement (Using GPC)

**Figure 3:**
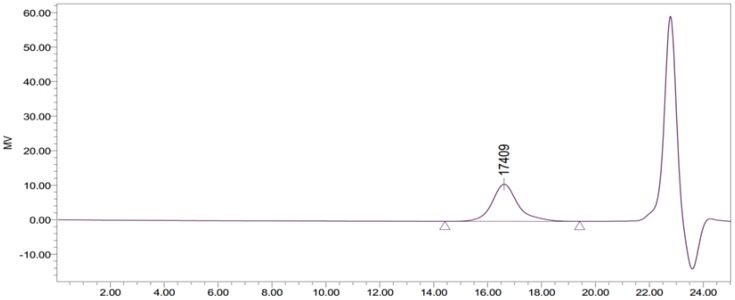
Gel permeation chromatogram of GO-ZnO-PLLA molecular weight.

The molecular weight of the GO-ZnO-PLLA polymer was measured by gel permeation chromatography (GPC) (Waters, Milford, MA, USA). The GO-ZnO-PLLA polymer had sharp, unimodal distributions, indicating that GO-ZnO had completely copolymerized with lactide and that no lactide homopolymerization had occurred. The standard was polystyrene, and the mobile phase was tetrahydrofuran (THF). The number average molecular weight (Mn) of polymerized poly(l-lactide)-grafted graphene oxide (GO-ZnO-PLLA) was 15.8 thousand, with a polydispersity of 1.10 (Figure 3 and Table 1). The ring-opening polymerization of surface l-lactide was achieved, as indicated by the detection of only one peak in the GPC analysis. The molecular weight and polydispersity were narrow and even.

### 3.3. X-ray Photoelectron Spectroscopy (XPS) Analysis of GO-ZnO-PLLA

**Figure 4:**
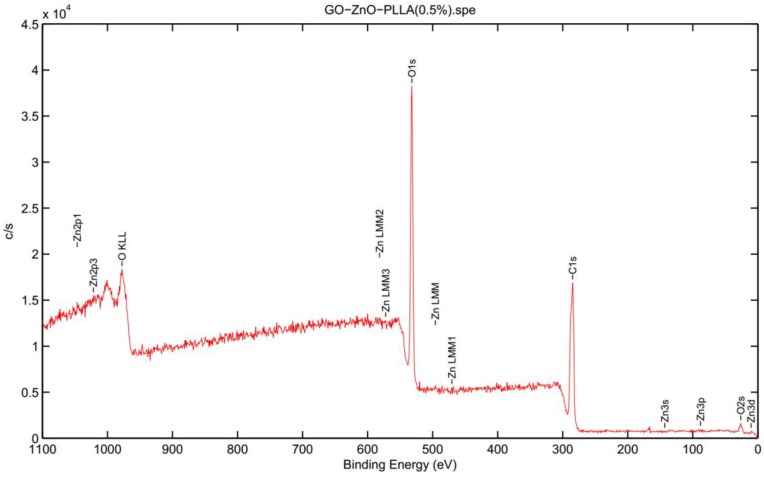
XPS (X-ray Photoelectron Spectroscopy) survey spectrum of GO-ZnO-PLLA.

Figure 4 shows that the X-ray photoelectron spectroscopy (XPS) obtains spectra by radiating samples using X-rays, which excite electrons from the inner shells or the valence shell, and then analyzing the energy of the emitted photoelectrons. Each element has its own characteristic spectrum, which allows qualitative analysis of sample elemental composition. Figure 4 illustrates the XPS survey spectrum of GO-ZnO-PLLA. The peaks in the figure were attributed to the elements Zn, O, and C; there were no characteristic peaks for other elements, further proving the presence of GO-ZnO-PLLA composite. The results suggested that PLLA was successfully grafted onto the surface of GO-ZnO.

### 3.4. Infrared (IR) Spectroscopy

**Figure 5:**
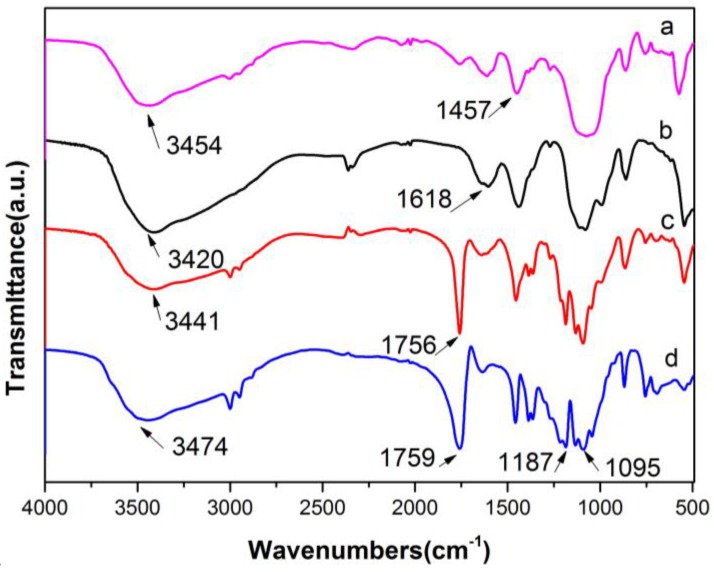
Fourier transform IR (FTIR) spectra of (**a**) PLLA, (**b**) GO-ZnO, (**c**) GO-Zn**O**/PLLA, and (**d**) GO-ZnO-PLLA.

Figure 5 shows the Fourier transform IR (FTIR) spectra of the polymers, in which the typical polyester absorption peaks appeared. In addition, the PLLA, GO-ZnO, GO-ZnO/PLLA, and GO-ZnO-PLLA IR measurements detected changes in the chemical functional groups upon grafting, as shown. The peaks at 3454 cm^−1^, 3420 cm^−1^, 3441, and 3474 cm^−1^ belong to the O–H stretching peaks in PLLA, GO-ZnO, GO-ZnO/PLLA, and GO-ZnO-PLLA, respectively. The peak at 1759 cm^−1^ corresponds to the stretching vibration peak of C=O in the ester bond in the PLLA chain (Figure 5a,d) [26,27]. A significantly stronger C=O stretching vibration peak appeared at 1759 cm^−1^ for GO-ZnO-PLLA, which was likely caused by the grafting of the PLLA molecular chains onto the surface of GO-ZnO. Therefore, the hydroxyl groups on the surface of GO-ZnO initiated the ring-opening of the lactide and the esterification with carboxyl groups [26]. The GO-ZnO/PLLA blends were also tested by infrared contrast, and the GO-ZnO/PLLA curves of the blends were found to be different from that of the copolymer (Figure 5d), it is consistent with the findings of Sun and He [28], in which GO-ZnO-PLLA was prepared by the ring-opening polymerization of lactide. Furthermore, the characteristic peaks of PLLA, including the stretching vibration of C–CH_3_, the bending vibration of –CH_3_, and the asymmetric bending vibration of –CH_3_, appeared at 1095, 1187, and 1457 cm^−1^, respectively [29,30,31,32,33], in the spectrum of GO-ZnO-PLLA. The results thus show that the PLLA molecular chains were successfully grafted onto the surface of GO-ZnO.

### 3.5. Differential Scanning Calorimetry (DSC) of PLLA and Its Composites

**Figure 6:**
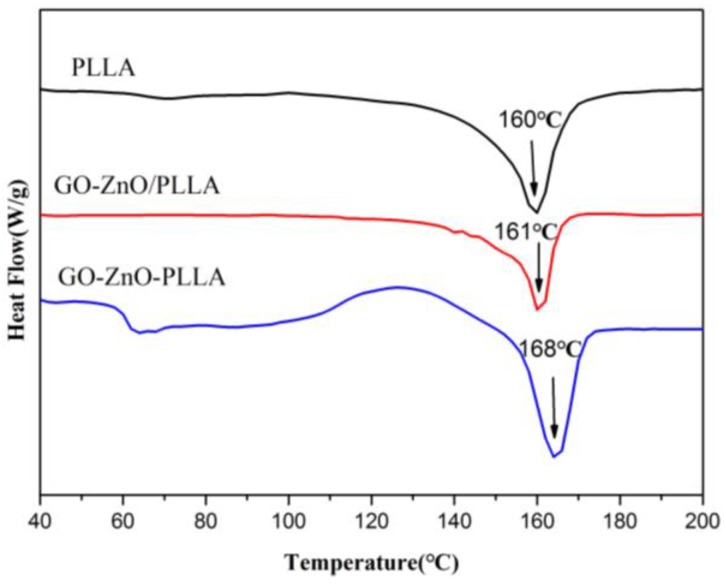
Differential scanning calorimetry (DSC) curves of PLLA and PLLA and its composites.

Figure 6 shows the differential scanning calorimetry (DSC) of PLLA and its composites. The melt crystallization behavior was investigated by DSC measurements, DSC cooling scan thermogram of PLLA (Mn¼ 20,000), PLLA, GO-ZnO-PLLA, and GO-ZnO/PLLA blend at the cooling rate of 10 C/min. A neat PLLA crystallizes in a broad crystallization temperature range with peak crystallization appearing at 160 °C. The crystallization peak temperature of PLLA in GO-ZnO/PLLA occurs at 161 °C, and the temperature range of crystallization is broad, while the crystallization temperature of the GO-ZnO-PLLA shifts to the higher temperature at 168 °C and the temperature range becomes narrow.

The nonisothermal crystallization behavior of GO-ZnO/PLLA blend, which has the same composition of GO-ZnO as that of GO-ZnO-PLLA, was performed to compare with GO-ZnO-PLLA. The crystallization temperature of PLLA in the GO-ZnO/PLLA is about 161 °C. As compared with neat PLLA, the crystallization temperature of PLLA in the GO-ZnO/PLLA has not been increased. However, the crystallization temperature of PLLA in the GO-ZnO-PLLA was increased to 168 °C., suggesting that the GO-ZnO platelets incorporated into the PLLA matrix have a nucleating effect on the crystallization of PLLA in the GO-ZnO-PLLA. Based on the comparison between the results of GO-ZnO-PLLA and GO-ZnO/PLLA blend, a nucleating effect found in the melt crystallization of GO-ZnO-PLLA is not only due to the individual GO-ZnO platelets in the PLLA matrix, but is also due to the tight bonding between the GO-ZnO platelets [25]. 

### 3.6. Thermogravimetric Analysis (TGA)

**Figure 7:**
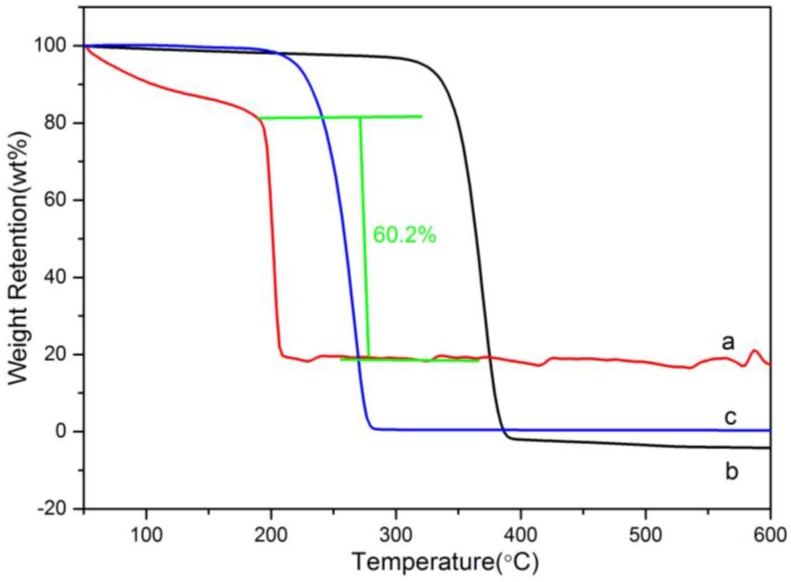
TGA (Thermogravimetric Analysis) curve of (**a**) GO-ZnO, (**b**) PLLA, and (**c**) GO-ZnO-PLLA.

As observed in Figure 7, the mass loss zone for GO-ZnO was 50–200 °C. The loss at 50–100 °C was attributed to the physical evaporation of water from GO of GO-ZnO. The loss at 100–200 °C was attributed to the decomposition of the functional groups, such as hydroxyl and carboxyl groups, on the GO-ZnO surface that decomposed to CO, CO_2_, and water vapor [34,35]. The mass loss of GO-ZnO-PLLA at 220–280 °C could be divided into two segments. At 220–250 °C, the mass loss was attributed to the decomposition of residual oxygen-containing functional groups; at 250–280 °C, the mass loss was attributed to the decomposition of PLLA grafted onto Go-ZnO [36]. The mass loss of PLLA mostly occurred at 340–380 °C, while the mass loss of GO-ZnO-PLLA was between those of GO-ZnO and PLLA. These results suggested that the PLLA molecular chains were successfully grafted onto the GO-ZnO surface. Based on the literature and according to the TGA curves, the grafting ratio of PLLA to the surface of GO-ZnO was approximately 60.2 wt % [37] for the in-situ ring-opening polymerization of the lactide. The weight loss of GO-ZnO-PLLA was between those of GO-ZnO and PLLA. We also performed a TG analysis on GO-ZnO-PLLA blends, and the results showed that a significant difference existed compared to the copolymer of GO-ZnO-PLLA. The results thus show that the PLLA molecular chains were successfully grafted to the surface of GO-ZnO.

### 3.7. Physical Property Characterization

**Figure 8:**
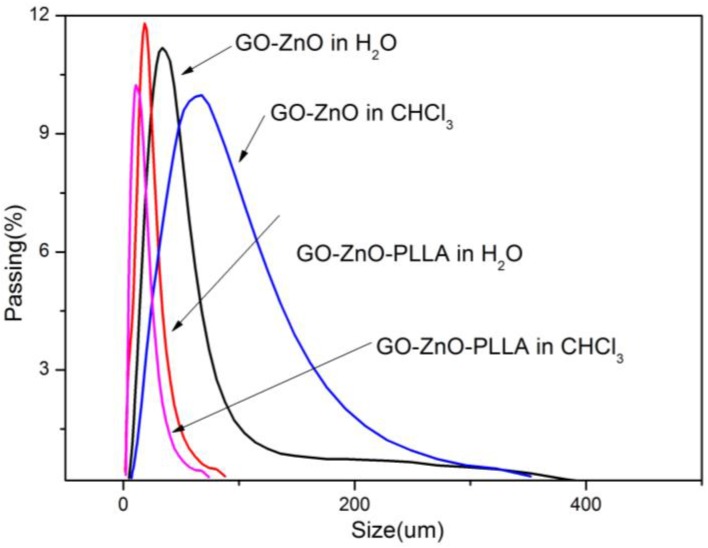
Laser analysis of particle size and shape of GO-ZnO and GO-ZnO-PLLA.

To further characterize the dispersity of GO-ZnO and GO-ZnO-PLLA in solution before and after grafting, we performed dynamic light scattering (DLS) to test the particle size and distribution of the materials in solution. The solutions were all at a concentration of 1 mg/mL. The materials were dissolved and ultrasonicated for 2 h prior to a rapid analysis. The results of the analysis are illustrated in Figure 8. Before grafting, the particle diameter of GO-ZnO was 31.01 μm in H_2_O and 55.56 μm in chloroform. The dispersity of GO-ZnO in chloroform was significantly better than that in water. The data suggested that GO-ZnO existed stably in water in the form of single-layer or few-layers [36]. There was a strong Van der Waals force and the movement of a large molecular chain, such as a bond or electrostatic attraction, between the chains between GO nanolayers, so the layers tended to aggregate. Hence, the particle size and particle size distribution could reflect the dispersity of the material in solvents [38]. The particle size of GO-ZnO-PLLA in water was 15.14 μm and gave a wide distribution of particle size. These results showed that after lactide ring-opening polymerization, the hydrophobic PLLA was successfully grafted onto the surface of GO, which then turned GO-ZnO-PLLA into a hydrophobic material and reduced its dispersity in water. The particle size in chloroform was 10.57 μm, suggesting that the dispersity of GO-ZnO-PLLA in chloroform was far greater than that in water. After grafting, GO changed from a hydrophilic material to a hydrophobic material. Thus, it is feasible to increase the dispersity of functionalized GO-ZnO-PLLA in chloroform by triggering lactic acid anhydride via the hydroxyl group on the GO surface.

### 3.8. Dispersions of GO-ZnO–PLLA and GO-ZnO/PLLA in Chloroform

**Figure 9:**
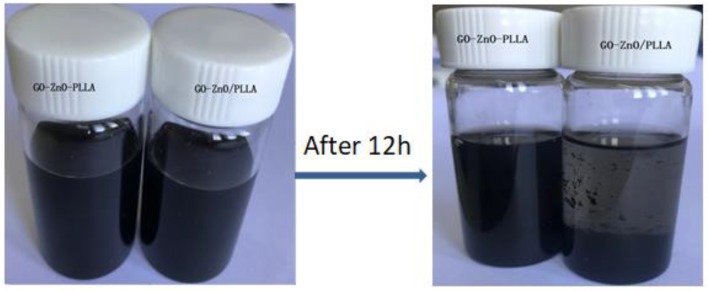
Dispersions of GO-ZnO-PLLA and GO-ZnO/PLLA in chloroform.

To further confirm the solubility of GO-ZnO/PLLA and GO-ZnO-PLLA in chloroform, we prepared 0.5 mg/mL GO-ZnO/PLLA and GO-ZnO-PLLA solutions in chloroform. After the solutions were sonicated for 2 h and left standing for 12 h (Figure 9), the solubility was investigated. All the GO-ZnO/PLLA precipitated to the bottom of the chloroform after the solution was sonicated and left for 12 h, whereas GO-ZnO-PLLA was still evenly dispersed in the chloroform. Similarly, when chloroform was used to dissolve GO-ZnO/PLLA and PLLA blends, 12 h later, GO-ZnO/PLLA was precipitated, because the dispersion of GO-ZnO/PLLA in chloroform is poor. These different behaviors indicate that the PLLA molecular chains grafted to the GO-ZnO/PLLA surface very strongly interacted with the solvent, thus increasing the dispersion of GO-ZnO-PLLA in chloroform [37].

### 3.9. Testing of Tensile Properties of PLLA and GO-ZnO-PLLA/PLLA Composite Films

**Figure 10:**
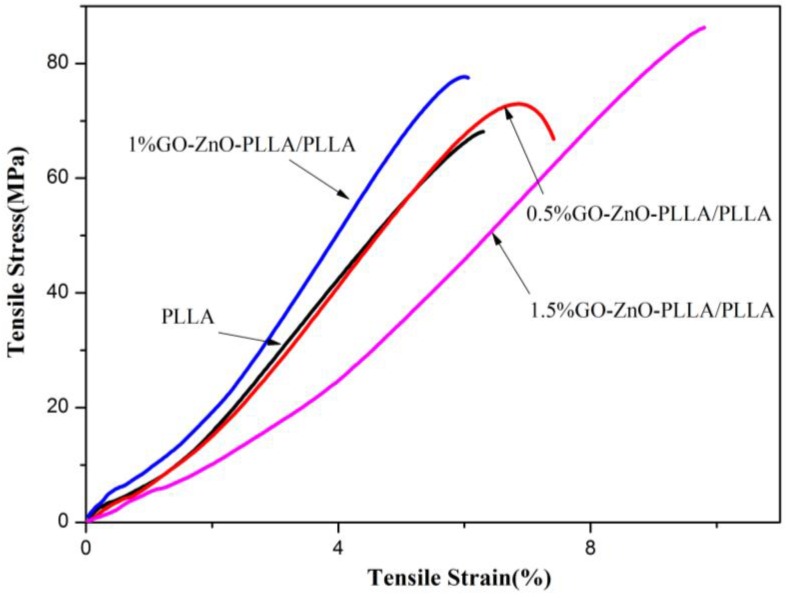
The stress–strain curve of PLLA and GO-ZnO-PLLA/PLLA composite films.

Figure 10 and Table 2 show the changes in the tensile strength and the elongation at break of PLLA and GO-ZnO-PLLA/PLLA composite films. All film samples tested were 1.5 mm in thickness and 15 mm in width. The results of the tensile property test showed that for pure PLLA, the tensile strength was 68.12 MPa, and the elongation at break was 6.3%. With the addition of 0.5% GO-ZnO-PLLA, the tensile strength and elongation rate of PLLA increased to 72.94 MPa and 7.42%, respectively. With the addition of 1.5% of the composite material GO-ZnO-PLLA, the tensile strength and elongation rate increased to only 86.24 MPa and 9.8%, respectively. These results suggested the GO-ZnO-PLLA composite could strengthen PLLA. Compared with pure PLLA, the tensile strength and the elongation rate of the PLLA with 1.5% GO-ZnO-PLLA composite increased by 26.6% and 55.5%, respectively. These results supported the findings in the literature that graphene and its derivatives have higher specific surface area and higher elastic modulus, and the dispersion of these materials in polymers can significantly enhance the load-carrying capability of polymers [39,40,41]. Table 2 compares the elongation at break of the composite film; it can be found that the elongation at break of the composite film decreases with addition of 1.0% GO-ZnO-PLLA, which may be due to the strong interface between GO-ZnO-PLLA and polylactic acid that limits the movement of polylactic acid molecular chain to a certain degree [42], so its elongation at break is relatively reduced. Additionally, there are some small differences.

### 3.10. SEM Analysis of GO-ZnO-PLLA/PLLA

**Figure 11:**
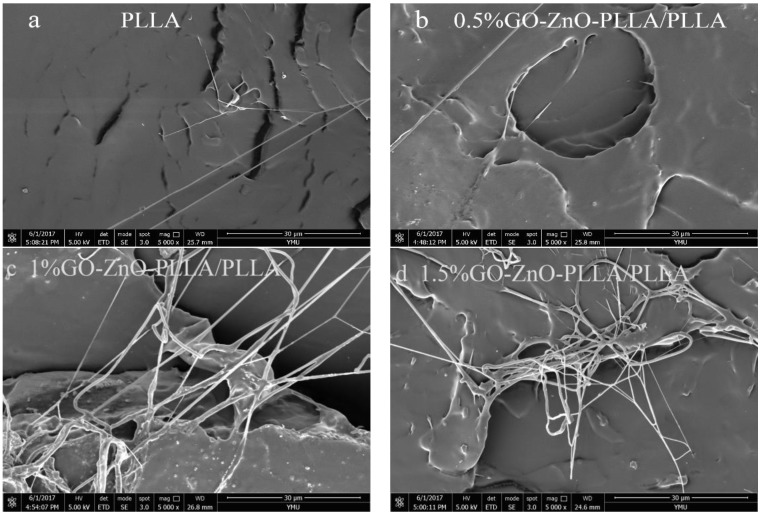
SEM (scanning electron microscopy) of fracture surfaces of PLLA and GO-ZnO-PLLA/PLLA composite films.

To observe the dispersity of GO-ZnO-PLLA in the composite material and the fracture surface morphology, we employed scanning electron microscopy (SEM) to examine the fracture surfaces of PLLA and GO-g-PLLA/PLLA composite films (Figure 11). Figure 11a illustrates the SEM photo of fracture surfaces of pure PLLA and GO-ZnO-PLLA/PLLA composite films. As seen in Figure 11a, the fracture surface of PLLA was neat and smooth. Obvious bumps were not observed in large numbers. The fracture surfaces of GO-ZnO-PLLA/PLLA composite films illustrated in Figure 11b–d were rougher and denser than those of PLLA, thereby requiring higher energy at fracture than pure PLLA. The fracture type was also different from that of pure PLLA, which showed obvious brittle fracture. On the other hand, there was no apparent graphene clustering observed on the fracture surfaces, indicating that GO-ZnO-PLLA was evenly distributed in PLLA. When 0.5 wt %, 1 wt % and 1.5 wt % of GO-ZnO-PLLA were added to PLLA, it was clearly seen that the GO-ZnO-PLLA flakes were tightly embedded in PLLA and showed strong interface adhesion with PLLA.

### 3.11. XRD Analysis of the GO-ZnO-PLLA/PLLA Composite Film

**Figure 12:**
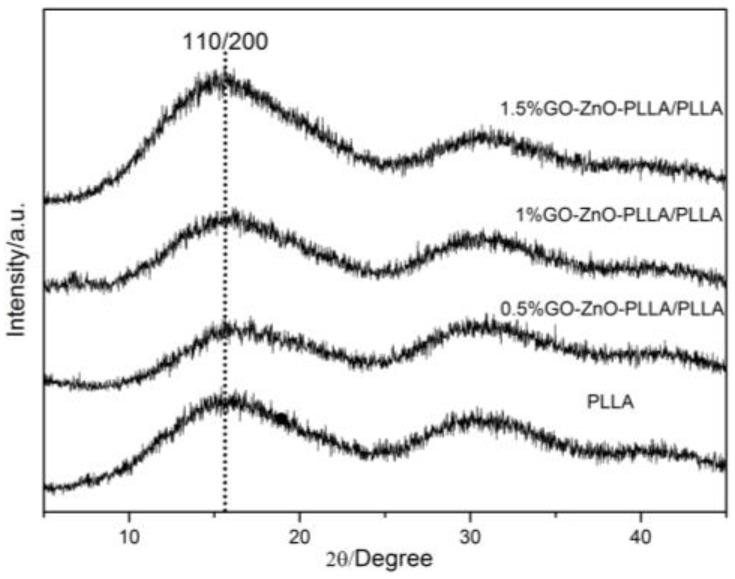
The XRD patterns of pure poly(lactic acid) film and its GO-ZnO-PLLA/PLLA composite film.

Figure 12 illustrates the XRD patterns of pure PLLA film and its GO-ZnO-PLLA/PLLA composite film. As seen, the most intense diffraction peak at 2θ = 16.9° corresponded to the PLLA (110) and (200) crystal PLLAnes; the peaks in the figure all belonged to the α crystalline phase of PLLA. For the GO-ZnO-PLLA/PLLA composite film, the location and shape of the diffraction peaks of the three samples were similar to those of pure PLLA. This similarity suggests that the addition of GO-ZnO-PLLA did not cause significant changes in the crystal structure of PLLA. Additionally, XRD of the composite films did not indicate a characteristic peak for GO at approximately 2θ = 10.5°, nor did it reveal that of nano-ZnO. These results suggest that the growth of nano-ZnO disturbed the ordered and layered structure of GO and caused the stripping of the GO layers, which led to the disappearance of characteristic peaks [43,44,45]. Moreover, GO-ZnO-PLLA was evenly distributed in the PLLA matrix, and many GO aggregates were not observed. As learned from the intensity of the diffraction peaks, the peak of the composite film at approximately 16.9° was significantly enhanced when 1.5 wt % of GO-ZnO-PLLA was added. When the GO-ZnO-PLLA load was increased, the diffraction peak became sharper, and the intensity was higher, indicating that the crystallization capacity of the composite film was enhanced.

### 3.12. Test of the Antibacterial Properties of GO-ZnO-PLLA/PLLA Composite Film

(1) Antibacterial test: A bacterial suspension (0.2 mL) was dropped onto film samples with each examined composition. For films of each composition, three sets of tests were performed in parallel. Sterilized polyethylene films were picked up and applied to the respective samples using sterilized tweezers. The coverage by polyethylene film ensured that the tested bacterial suspension was completely covered and allowed even distribution of the bacterial suspension on the sample surfaces. The samples were PLLAced on sterilized petri dishes and cultured at 37 ± 1 °C and relative humidity (RH) > 90% with and without light exposure for 24 h. This improvement may be because the hole (h+) reacts with OH− on the surface of ZnO nanoparticles, resulting in the generation of reactive oxygen species such as hydroxyl radicals (OH^•^), superoxide anion (O_2_^−^) and perhydroxyl radicals (HO_2_^•^), which lead to the decomposition and damage of bacterial cells [46].

(2) Colony count: The cultured samples were removed from the dishes and added to 20 mL of sterilized saline to elute the bacteria solution. The eluted bacterial solutions were adequately mixed. The live bacteria counts in the bacterial suspensions after antibacterial treatment were determined by the colony counting method in GB/T4789.2-2010.

The formula for calculating the antibacterial rate is as follows:

R (%) = (B − C)/B × 100(1)

in which R is the antibacterial rate (%), B is the average recovered bacteria count from the pure PLLA film sample (cfu/film), and C is the average recovered bacteria count from the PLLA film samples added with antibacterial components (cfu/film). The test results are given in Table 3 and Table 4.

**Figure 13:**
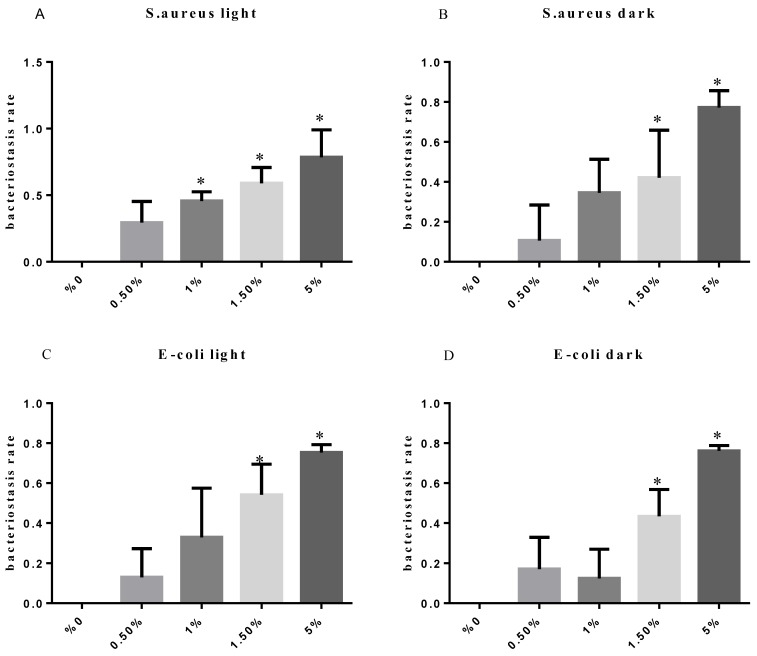
Statistical plots of the antibacterial rates of (**A**) inhibition of samples against S. aureus under light exposure, (**B**) inhibition of samples against *E. coli* under light exposure, (**C**) inhibition of samples against *S. aureus* without light exposure, and (**D**) inhibition of samples against *E. coli* without light exposure. * indicates a significant difference with 0% (PLLA), *p* < 0.05.

Table 3 and Table 4, and Figure 13 show the test results of the antibacterial properties of GO-ZnO-PLLA/PLLA composite film, Figure 13A shows the inhibition of samples against *S. aureus* under light exposure. Compared with the group with 0% GO-ZnO-PLLA, the differences observed on the groups with 1%, 1.5% and 5% of GO-ZnO-PLLA were significantly different. These results indicated that inhibition of *S. aureus* was observed with only 1% of GO-ZnO-PLLA. The inhibition of samples against *E. coli* under light exposure. Compared with the group with 0% GO-ZnO-PLLA, the differences observed on the groups with 1%, 1.5%, and 5% of GO-ZnO-PLLA were significantly different. These results indicated that inhibition of *E. coli* was observed with only 1% of GO-ZnO-PLLA (Figure 13B). The inhibition of samples against *S. aureus* without light exposure (Figure 13C). The inhibition of samples against *E. coli* without light exposure (Figure 13D). Under light exposure, the antibacterial rate of the material with 1.5% of go-zno-pllareached 42.1% and 43.3% against *S. aureus* and *E. coli*, respectively. When 5% of antibacterial component was added, the antibacterial rates against *S. aureus* and *E. coli* reached 78.5% and 75.3%, respectively. Without light exposure, the material with 1.5% of antibacterial component showed a relatively significant antibacterial property. The antibacterial rate reached 58.9% and 54.1% against *S. aureus* and *E. coli*, respectively. When 5% of antibacterial component was added, the antibacterial rates against the two bacteria reached 77.2% and 76.1%, respectively.

## 4. Conclusions

GO was employed for the preparation of GO-ZnO. The hydroxyl group on the surface was exploited to trigger the l-lactide ring-opening polymerization. A GO-ZnO-PLLA composite material with grafting of the PLLA chain to the GO-ZnO surface was prepared. The chemical structure of the GO-ZnO surface was changed with improved dispersion of GO-ZnO in organic solvents, further enhancing the GO-ZnO dispersion in PLLA matrix and its interface bonding with PLLA. This preparation method greatly improved the compatibility of the functionalized material. The obtained material had higher specific surface area, higher elastic modulus and enhanced load-carrying capability. The GO-ZnO-PLLA/PLLA composite film had excellent antibacterial properties. With the addition of 1.5% of GO-ZnO-PLLA, the antibacterial function was more prominent under light exposure than without light exposure.

## Figures and Tables

**Table 1:** Results of gel permeation chromatogram analysis of GO-ZnO-PLLA molecular weight.

Mn	Mw	MP	Mz	Mz + 1	Polydispersity (Mw/Mn)	Mz/Mw
15,784	17,362	17,409	18,912	20,584	1.099972	1.08925

**Table 2:** Test of tensile properties of PLLA and GO-ZnO-PLLA/PLLA.

Sample Name	Elastic Modulus (MPa)	Elongation at Break (%)	Tensile Stress at Break (MPa)	Tensile Strength (MPa)
PLLA	1356.6	6.3	68.12	68.12
(0.5%) GO-ZnO-PLLA/PLLA	1405.58	7.42	66.83	72.94
(1.0%) GO-ZnO-PLLA/PLLA	1668.62	6.86	77.49	77.65
(1.5%) GO-ZnO-PLLA/PLLA	1730.62	9.8	86.24	86.24

**Table 3:** Test results of the antibacterial properties of GO-ZnO-PLLA/PLLA composite film under light exposure.

Samples	*Staphylococcus aureus* (*S. aureus*)	-	*Escherichia coli* (*E. coli*)	-
-	Colony Photo	Antibacterial Rate R/%	Colony Photo	Antibacterial Rate R/%
PLLA	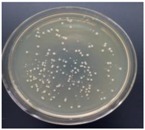	-	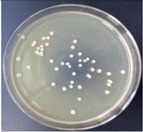	-
0.5% GO-ZnO-PLLA	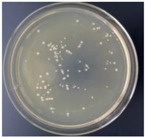	29.4 ± 5.3	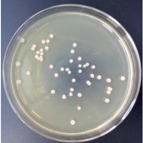	12.9 ± 4.7
1% GO-ZnO-PLLA	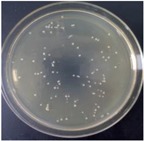	45.7 ± 2.3	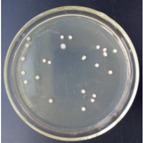	32.9 ± 8.1
1.5% GO-ZnO-PLLA	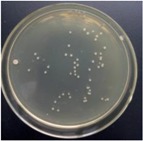	58.9 ± 3.9	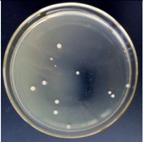	54.1 ± 5.1
5% GO-ZnO-PLLA	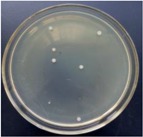	78.5 ± 6.8	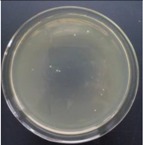	75.3 ± 1.3

**Table 4:** Test results of the antibacterial properties of GO-ZnO-PLLA/PLLA composite film without light exposure.

Sample	*Staphylococcus aureus* (*S. aureus*)	-	*Escherichia coli* (*E. coli*)	-
-	Colony Photo	Antibacterial Rate R/%	Colony Photo	Antibacterial Rate R/%
PLLA	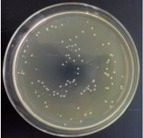	-	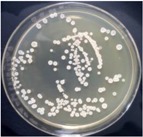	-
0.5% GO-ZnO-PLLA	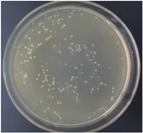	10.7 ± 5.9	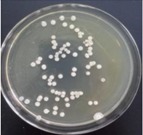	17.1 ± 5.2
1% GO-ZnO-PLLA	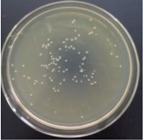	34.5 ± 5.5	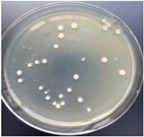	12.4 ± 4.8
1.5% GO-ZnO-PLLA	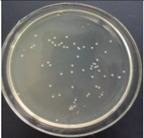	42.1 ± 7.9	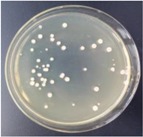	43.3 ± 4.5
5% GO-ZnO-PLLA	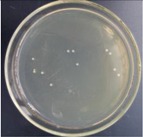	77.2 ± 2.8	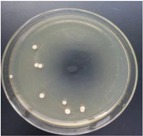	76.1 ± 0.9
